# A Multiplexable Op-Amp Interface for Accurate Readout of Remote Resistive Sensors

**DOI:** 10.3390/s26020461

**Published:** 2026-01-10

**Authors:** Sanya Kuankid, Jirapong Jittakort, Apinan Aurasopon

**Affiliations:** 1 Department of Electrical Engineering and Technology, Faculty of Science and Technology, Nakhon Pathom Rajabhat University, Nakhon Pathom 73000, Thailand; sanya@webmail.npru.ac.th; 2 Department of Electrical Engineering, Faculty of Technical Education, Rajamangala University of Technology Thanyaburi, Pathum Thani 12110, Thailand; jirapong_j@rmutt.ac.th; 3 Faculty of Engineering, Mahasarakham University, Mahasarakham 44150, Thailand

**Keywords:** resistive sensors, two-wire configuration, op-amp interface, diode steering, square-wave excitation, lead-wire compensation, sensor multiplexing, remote sensing, voltage plateau sampling

## Abstract

This paper presents a compact and accurate readout circuit for remote two-wire resistive sensors, based on an inverting operational amplifier with a fixed bias voltage, diode steering, and unidirectional square-wave excitation generated by a microcontroller. The proposed method determines the sensor resistance by directly sampling two steady-state voltage plateaus at the op-amp output during alternating excitation phases. This approach enables fast, lead-wire-insensitive measurements without the need for analog filtering or precise PWM duty-cycle control. The architecture supports sensor array multiplexing via analog switches, allowing scalable, low-power implementation. Experimental results demonstrate a maximum relative error of 0.23% across a wide resistance range (0.5–3.5 kΩ), confirming the method’s suitability for low-cost, embedded, and remote sensing applications.

## 1. Introduction

Resistive sensors are widely adopted due to their simple structure, low cost, and compatibility with digital electronics. These sensors operate by converting physical quantities—such as temperature, pressure, humidity, or gas concentration—into a corresponding change in electrical resistance. Common examples include thermistors, strain gauges, metal-oxide gas sensors (e.g., MQ series), and resistive soil moisture probes [1,2]. Their versatility has led to broad applications in fields such as wearable health monitoring, smart agriculture, robotics, and environmental sensing [3,4]. For instance, textile-based resistive sensors are used in wearable systems to detect posture, motion, and respiration [3], while resistive moisture and gas sensors are commonly deployed in agricultural environments to monitor soil hydration and livestock air quality [1,4]. These widespread applications have increased the demand for compact, low-power, and accurate readout systems that can support reliable resistive sensing under noisy, mobile, or remote conditions.

While resistive sensors provide numerous advantages, their measurement accuracy is often compromised by parasitic resistances introduced by long interconnect wires—particularly in remote or distributed sensing scenarios. In two-wire configurations, the same pair of conductors is used for both excitation and signal acquisition, making it difficult to distinguish the sensor resistance from the resistance of the lead wires. This results in systematic measurement errors that increase with wire length and vary with temperature-induced changes in wire resistance [5]. To mitigate lead-wire resistance effects, three-wire and four-wire measurement techniques are commonly employed. The three-wire configuration relies on matched lead resistances and differential sensing to compensate for one wire contribution, whereas the four-wire (Kelvin) method completely separates the excitation and sensing paths, enabling highly accurate measurements even over long cable lengths. Despite their effectiveness, both approaches require additional wiring and hardware resources, which increases system complexity and cost. As a result, they are poorly suited for compact, wearable, or large-scale deployments where many sensing points must be addressed. In parallel, alternative solutions have been proposed for direct microcontroller interfacing or simplified analog front-end implementation, typically based on resistance-to-voltage (R-to-V) or resistance-to-time (R-to-T) conversion techniques [6,7,8,9,10]. These methods translate resistance variations into voltage levels or timing intervals that can be readily digitized, reducing the need for dedicated instrumentation circuits. However, their performance is often constrained by nonlinearity, limited dynamic range, and sensitivity to parasitic elements such as switch resistance, comparator thresholds, and ADC nonidealities. More importantly, these approaches are difficult to extend to multi-sensor systems—such as electronic noses, tactile arrays, or distributed environmental sensors—where scalable, accurate readout with shared wiring and switching resources is required.

To address the limitations of conventional multi-wire configurations and direct R-to-V interface methods in multi-sensor systems, this work presents a diode-guided, op-amp-based readout circuit for two-wire resistive sensors using unidirectional square-wave excitation generated by a microcontroller. Unlike [11], which requires analog filtering and strict 50% PWM excitation, the proposed technique enables real-time resistance extraction by directly sampling two steady-state output voltage levels—simplifying both hardware and timing requirements. The core challenge of lead-wire and analog switch resistance is resolved by exploiting symmetric diode-steered conduction paths. During alternating excitation phases, the excitation current flows through mirrored paths that include both the sensor and its associated parasitic elements. As a result, undesired resistive voltage drops are inherently canceled when the resistance is computed from the differential output. This compensation is achieved without additional calibration or compensation circuitry. The proposed architecture supports fast response and improved scalability while maintaining a simple implementation based on a single operational amplifier and a row–column analog switch matrix. Sensor resistance is calculated from the op-amp output voltage plateaus relative to a virtual bias point, avoiding cumulative errors commonly introduced by analog averaging or comparator delays. Experimental results validate the effectiveness of the method over a wide resistance range from 0.5 to 3.5 kΩ, with a maximum relative error of only 0.23% even under significant lead-wire resistance. These characteristics make the proposed solution well suited for compact, low-power, and cost-sensitive sensing applications in remote, wearable, or distributed environments.

The remainder of the paper is organized as follows: Section 2 introduces the proposed configuration for lead-wire compensation and outlines the theoretical framework for resistance extraction. Section 3 presents experimental results, including waveform observations and measurement accuracy under various parasitic conditions. Section 4 provides a detailed discussion of the findings, comparing the proposed approach with existing resistive sensor readout techniques. Finally, Section 5 concludes the paper by summarizing the key contributions and practical implications of the method.

## 2. The Basis of the Proposed Method and Theoretical Analysis

The proposed method extends the diode-steered, op-amp-based configuration from [11], originally designed for accurate two-wire resistance measurement using bidirectional square-wave excitation. While effective for a single sensor, that approach lacks scalability and requires low-pass filtering and fixed-duty control. To address these limitations, our previous work [12] introduced a resistive sensor array interface using differential sampling and multi-phase averaging. Building on these foundations, the present system employs unidirectional square-wave excitation and a row-selectable analog switch matrix to dynamically insert each sensor into the feedback loop of a shared op-amp. This configuration enables fast, filter-free, and scalable two-wire measurements suitable for sensor arrays, while inherently compensating for parasitic wire and switch resistance.

### 2.1. Proposed Configuration

**Figure 1:**
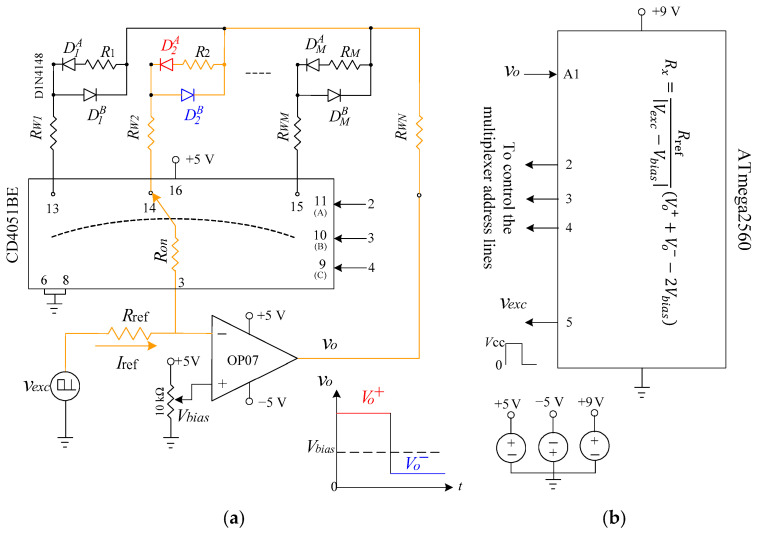
Proposed readout architecture for multiplexed resistive sensors. (**a**) Circuit configuration employing an inverting op-amp, diode steering, and unidirectional square-wave excitation. Each sensor is selectively inserted into the feedback path via analog switch, enabling accurate two-wire measurement. The op-amp output alternates between two voltage plateaus, $V_{o}^{+}$ and $V_{o}^{-}$, depending on the conduction phase. (**b**) Microcontroller implementation, where the digital output generates the excitation signal $v_{exc}$, the ADC samples the op-amp output $v_{o}$ during both excitation phases, and the select lines address individual sensors. The sensor resistance $R_{x}$ is computed digitally from the sampled plateau voltages.

The proposed circuit provides a scalable and accurate readout solution for multiplexed two-wire resistive sensors using a single inverting operational amplifier, diode steering, and unidirectional square-wave excitation. As illustrated in Figure 1a, a unipolar square-wave excitation voltage $v_{exc}$, toggling between 0 V and 5 V, is generated by a microcontroller output and applied across a precision reference resistor $R_{\mathrm{ref}}$. This produces a constant excitation current $I_{\mathrm{ref}}$, which serves as the stimulus for all sensor measurements. Sensor selection is achieved through an analog switch matrix that sequentially connects one sensor row to a shared column return line. Once a row is selected, the corresponding sensor is dynamically inserted into the feedback path of the operational amplifier. Each sensor node incorporates two diode paths ($D_{M}^{A}$ and $D_{M}^{B}$), which steer the excitation current depending on the state of $v_{exc}$. When $v_{exc}$ is high, one diode path conducts and the current flows through the sensor in one direction; when $v_{exc}$ is low, the complementary diode path conducts, reversing the sensor current. This mechanism enables effective bidirectional current excitation using a single unidirectional drive signal.

To ensure compatibility with single-supply microcontroller systems, the non-inverting input of the op-amp is biased to a fixed mid-supply voltage $V_{bias}$ (typically 2.5 V). This establishes a virtual reference point and forces the op-amp output to swing around $V_{bias}$, keeping both output levels within the ADC input range. During each excitation phase, the op-amp output settles to a stable voltage plateau—denoted $V_{o}^{+}$ or $V_{o}^{-}$—corresponding to the voltage drop across the active conduction path. Although the excitation current $I_{\mathrm{ref}}$ remains unidirectional, the diode-guided conduction paths cause the sensor current to alternate direction between phases. By sampling the two output plateaus and performing a differential calculation referenced to $V_{bias}$, the sensor resistance can be accurately extracted. This operation inherently cancels the effects of lead-wire resistance and analog switch on-resistance, without requiring analog low-pass filtering, signal averaging. As shown in Figure 1b, the microcontroller controls both the excitation signal $v_{exc}$ and the row-selection lines, while the ADC samples the op-amp output $v_{o}$ during each phase. The resistance computation is performed digitally, enabling a compact, low-power implementation suitable for multiplexed sensor systems.

### 2.2. Theoretical Derivation

**Figure 2:**
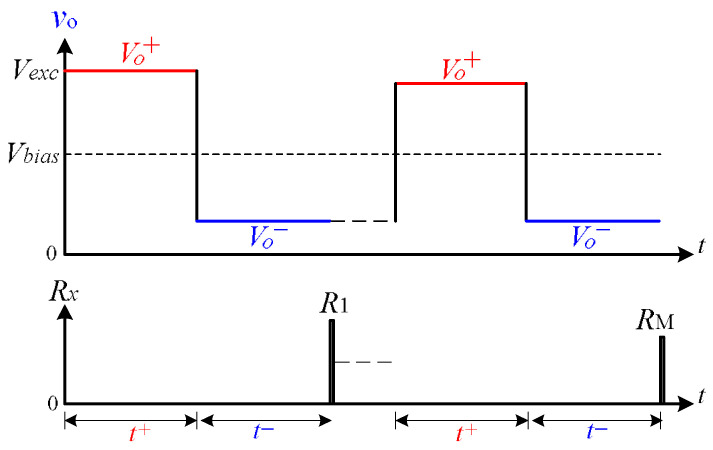
Measured op-amp output waveform $v_{o}$, illustrating voltage plateaus $V_{o}^{+}$ and $V_{o}^{-}$ during each excitation phase and multiplexed scanning across sensors $R_{1}$, $R_{M}$, etc.

The proposed circuit determines the sensor resistance by analyzing the steady-state output plateaus of the op-amp during two alternating excitation phases. As shown in Figure 2, a unipolar square-wave excitation signal $v_{exc}$ toggles between 0 V and *V*cc (e.g., 5 V), producing a constant excitation current $I_{\mathrm{ref}}=(v_{exc}-V_{bias})/R_{\mathrm{ref}}$ through the reference resistor $R_{\mathrm{ref}}$. Although this current remains unidirectional, the diode steering network causes the current through the sensor to alternate direction in each half-cycle.

Let us define:

- $V_{o}^{+}$: The op-amp output during the $t^{+}$ interval, when the sensor current flows in the reverse direction (e.g., through diode $D_{M}^{A}$). This corresponds to the lower excitation level $v_{exc}=0\;V$, causing the op-amp to drive current out.
- $V_{o}^{-}$: The op-amp output during the $t^{+}$ interval, when the sensor current flows in the reverse direction (e.g., through diode $D_{M}^{B}$). This corresponds to the lower excitation level $v_{exc}=5\;V$, causing the op-amp to drive current out.
- $R_{WM},\;R_{WN}$ are the lead-wire resistances and $R_{on}$ is the analog switch on-resistance.
- $V_{FA}$, $V_{FB}$ are diode forward voltages during each conduction path.

During the low phase of excitation ($v_{exc}=0\;V$), the excitation current is sourced from the op-amp output and flows through the path: op-amp →$\;R_{WN}\;$→$\;R_{x}\;$→$\;D_{M}^{A}\;$→$\;R_{WM}\;$→$\;R_{on}\;$→ $V_{bias}$.

Applying KVL, the op-amp output voltage becomes:

(1) $$V_{o}^{+}={V_{\mathrm{bias}}+I}_{\mathrm{ref}}\cdot \left(R_{WN}+R_{x}{+R}_{WM}{+R}_{on}\right)+V_{FA}$$

During the high phase of excitation ($v_{exc}=Vcc$), the current flows from $v_{exc}$ through $R_{\mathrm{ref}}$, into the op-amp (held at $V_{bias}$), and exits through the path: op-amp →$\;R_{WN}\;$→${\;D}_{M}^{B}\;$→${\;R}_{WM}\;$→${\;R}_{on}\;$→${\;V}_{bias}$.

Yielding:

(2) $$V_{o}^{-}={V_{\mathrm{bias}}-I}_{\mathrm{ref}}\cdot \left(R_{WM}+R_{WN}{+R}_{on}\right)-V_{FB}$$

It is important to note that the same analog switch channel is used during both excitation phases, such that the on-resistance $R_{on}$ appears identically in Equations (1) and (2). Consequently, $R_{on}$—along with the lead-wire resistances $R_{WM}$ and $R_{WN}$—acts as a common-path parasitic and does not introduce asymmetry between the two measurements. Assuming matched diode forward voltages ($V_{FA}\approx V_{FB}$) and constant parasitic elements, these terms cancel analytically when the differential output is formed. Moreover, while the CD4051B analog switch exhibits a nonlinear, voltage-dependent $R_{on}$ characteristic (e.g., ranging from ~125 Ω near 2.5 V to >300 Ω near 0 V or 5 V) [13], its effect is minimized by the symmetry of the current path and further mitigated through the differential measurement approach. Any residual nonlinearity is compensated by the one-point calibration and averaging method described in Section 3.2. The true sensor resistance $R_{x}$ can therefore be isolated as:

(3) $$R_{x}=\frac{R_{\mathrm{ref}}}{\left|v_{exc}-V_{bias}\right|}(V_{o}^{+}+V_{o}^{-}-2V_{bias})$$

### 2.3. Non-Ideality

Under non-ideal conditions, the resistance measurement using the proposed method may be affected by several error sources, including diode mismatch, op-amp input offset voltage ($V_{IO}$), input bias current ($I_{B}$), and ADC quantization. These effects contribute to offset and gain errors in the computed sensor resistance $R_{x}$, which are detailed below.

#### 2.3.1. Diode Forward Voltage Mismatch

In the ideal case, both steering diodes exhibit identical forward voltage drops, and the differential output voltage depends linearly on the sensor resistance $R_{x}$. However, in practice, a mismatch between the forward voltages of diodes $D_{M}^{A}$ and $D_{M}^{B}$ introduces a residual error. Let $∆V_{F}=V_{F_{B}}-V_{F_{A}}$, denote this mismatch. The total differential output over a full excitation cycle becomes:

(4) $$V_{o}^{+}+V_{o}^{-}-2V_{bias}=I_{\mathrm{ref}}\cdot R_{x,true}+∆V_{F}$$

As a result, the measured resistance becomes:

(5) $$R_{x,meas}=R_{x,true}+\frac{∆V_{F}}{I_{\mathrm{ref}}}$$

Since $∆V_{F}$ may be positive or negative, the resistance error can lead to either overestimation or underestimation. For example, if $\left|∆V_{F}\right|=0.2\;mV$ and $I_{\mathrm{ref}}=1\;mA$, the resulting resistance error is 0.2 Ω. For a 1 kΩ sensor, this corresponds to a 0.02% error. Using well-matched diodes or integrated diode pairs can significantly reduce this mismatch and improve overall accuracy.

#### 2.3.2. Op-Amp Voltage Offset

The input offset voltage of the op-amp introduces a consistent asymmetry in both measurement phases. During each half-cycle, the output voltage is shifted by ${\pm V}_{IO}$, depending on the direction of current flow. As a result, the total differential output is affected by a cumulative offset of ${2V}_{IO}$, leading to:

(6) $$V_{o}^{+}+V_{o}^{-}-2V_{bias}=I_{\mathrm{ref}}\cdot R_{x,true}+{2V}_{IO}$$

Solving for the measured resistance yields:

(7) $$R_{x,meas}=R_{x,true}+\frac{{2V}_{IO}}{I_{\mathrm{ref}}}$$

Assuming a typical op-amp input offset of $V_{IO}=\pm 300\;\mu V$ and an excitation current $I_{\mathrm{ref}}=1\;mA$, the resulting worst-case resistance error is 0.6 Ω. For a 1 kΩ sensor, this corresponds to 0.06% full-scale span (FSS). The error becomes proportionally more significant at lower excitation currents, making low-offset op-amps preferable in high-accuracy applications.

#### 2.3.3. Input Bias Current

The input bias current $I_{B}$ of the op-amp flows into or out of the inverting input, where the virtual ground is maintained at $V_{bias}$. In the proposed configuration, the inverting input node is connected to the column line through diode steering and the selected sensor path. As a result, any nonzero $I_{B}$ introduces a small additional current that either adds to or subtracts from the excitation current $I_{\mathrm{ref}}$, depending on its polarity.

(8) $$V_{o}^{+}+V_{o}^{-}-2V_{bias}={(I}_{\mathrm{ref}}{+I}_{B})\cdot R_{x,true}$$

Solving for the measured resistance:

(9) $$R_{x,meas}=R_{x,true}\cdot \left(1+\frac{I_{B}}{I_{\mathrm{ref}}}\right)$$

This results in a systematic underestimation of the true resistance $R_{x}$, since the denominator is larger than the nominal excitation current. For example, assuming $I_{\mathrm{ref}}=1\;mA$ and a typical op-amp input bias current $I_{B}=50\;nA$, the effective current increases by only 0.005%, resulting in a negligible error (about 0.5 Ω for a 10 kΩ sensor).

#### 2.3.4. ADC Nonlinearities

In the experimental setup, an ATmega2560 microcontroller was used to acquire the op-amp output voltage via its built-in 10-bit successive-approximation ADC. This ADC operates with a full-scale reference voltage of $V_{ref,ADC}=5V$, providing a digital output described by the expression [14]:

(10) $$V_{m}=D_{m}\cdot \frac{V_{FS}}{2^{10}}\beta +D_{offset}\cdot \frac{V_{FS}}{2^{n}}$$

where $D_{m}$ is the output code, β is the gain error coefficient, and $D_{\mathrm{offset}}$ is the offset code.

According to the ATmega2560 datasheet [15] (p. 382), the ADC exhibits a worst-case absolute accuracy of ±2.25 LSB under typical operating conditions, which includes the combined effects of gain error, offset error, differential nonlinearity (DNL), and integral nonlinearity (INL). For a 10-bit ADC with a full-scale voltage of 5 V, one LSB corresponds to approximately 4.88 mV, yielding a maximum voltage uncertainty of about 11 mV. The proposed readout method calculates sensor resistance from the differential quantity $V_{o}^{+}+V_{o}^{-}-2V_{\mathrm{bias}}$, which inherently cancels static gain and offset errors. However, DNL and quantization noise still introduce small deviations in the digitized values. Given the voltage-to-resistance scaling factor of approximately 800 Ω/V, this voltage uncertainty contributes a resistance error of around 8.8 Ω, or 0.35% over the 0.5–3.5 kΩ range. To further suppress ADC-induced errors, 100 samples are averaged during each excitation phase. This averaging reduces the influence of random quantization noise and DNL-related fluctuations by a factor of $1/\sqrt{N}$, lowering the effective ADC contribution to below 0.2% full-scale span (FSS). These results indicate that, while ADC nonlinearity represents a fundamental limitation, its impact on the proposed readout scheme is effectively mitigated through differential measurement and digital averaging.

## 3. Experimental Results

**Figure 3:**
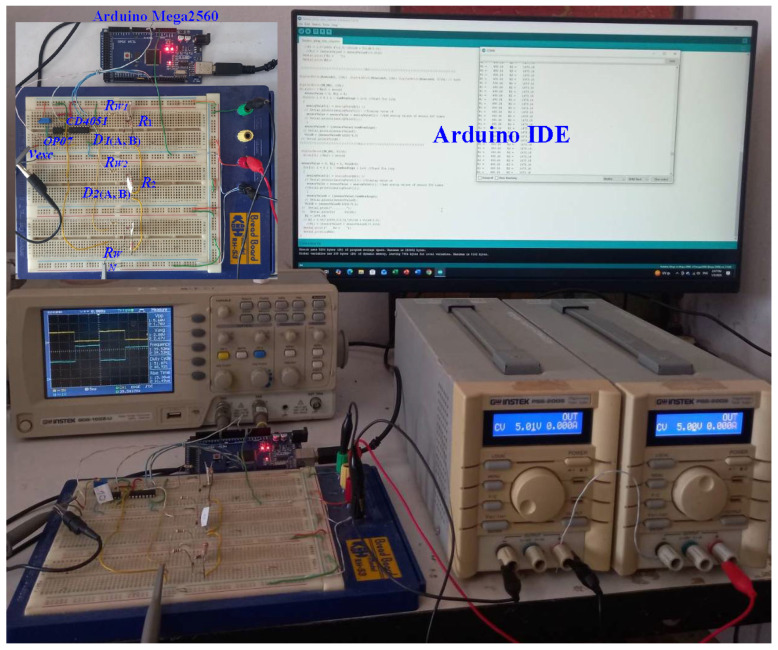
Experimental setup showing the Arduino Mega2560, OP07 op-amp, CD4051B multiplexer, ±5 V power supplies, and oscilloscope used to capture $v_{exc}$ and $v_{o}$ waveforms during sensor scanning.

The experimental system was built to validate the proposed resistive sensor array readout method. The main components and configuration details are summarized in Table 1. The setup emulates practical remote sensing conditions, including lead-wire parasitic resistance and time-multiplexed sensor scanning via analog switches. The Arduino Mega2560 microcontroller serves as the core controller, while an OP07 precision op-amp and CD4051B multiplexer form the analog front-end. Special attention was given to ensuring that the prototype reflects real-world, low-cost embedded deployments. This configuration allows systematic evaluation of measurement accuracy, linearity, and robustness to parasitic effects. A TECPEL DMM8050 multimeter was used to verify the actual resistor values used as sensor emulators. Figure 3 shows the full experimental setup, including the Arduino board, dual ±5 V power supplies, oscilloscope used to monitor waveforms, and the PC running the Arduino IDE to log computed resistance values.

### 3.1. Waveform Observation During Array Scanning

**Figure 4:**
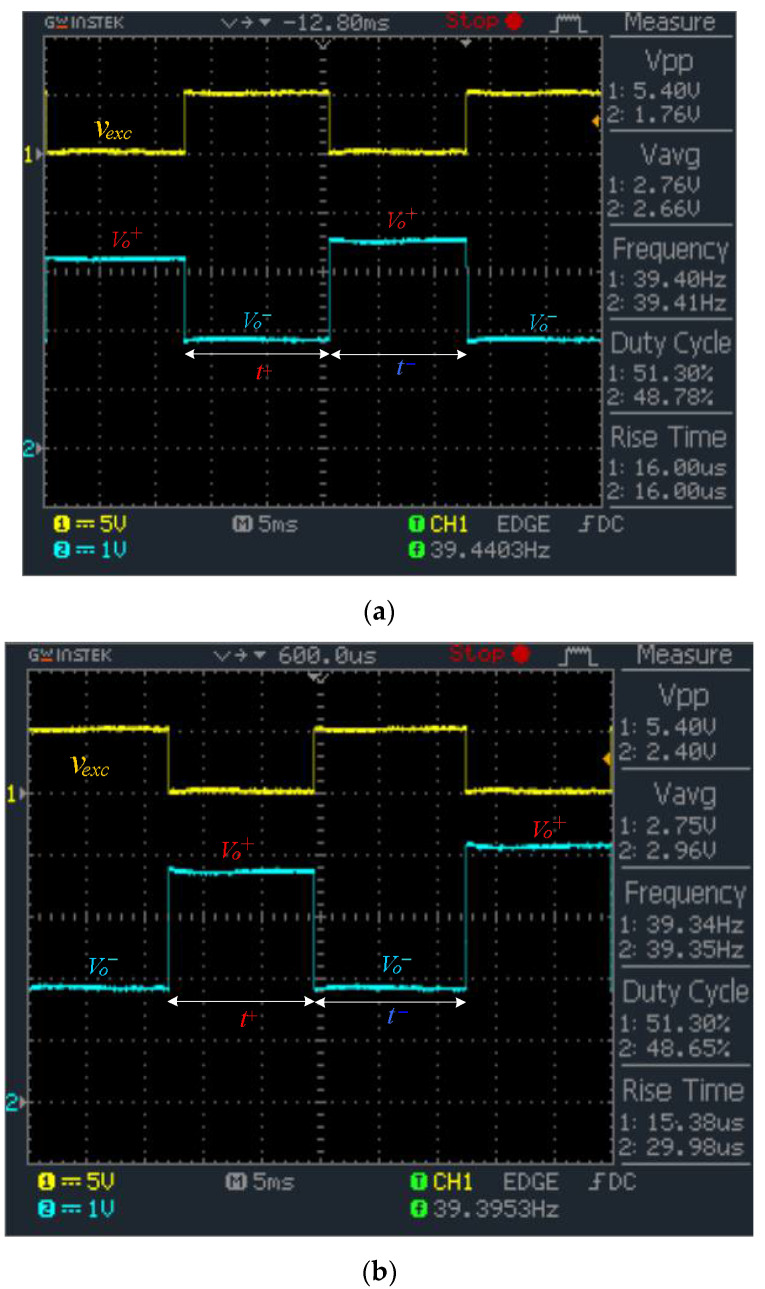
Oscilloscope waveforms of the excitation signal $v_{exc}$ (yellow trace) and the op-amp output waveform $v_{o}$ (blue trace) during sequential scanning of two resistive sensors. (**a**) $R_{x}$ = 0.49 kΩ and 1.469 kΩ; (**b**) $R_{x}$ = 2.18 and 3.47 kΩ.

To validate the switching behavior and output voltage response of the proposed system, the op-amp output $v_{o}$ was monitored in real time using an oscilloscope. Figure 4a shows the waveform corresponding to two sensor nodes with $R_{1}$ = 0.49 kΩ and $R_{2}$ = 1.469 kΩ, while Figure 4b presents results for $R_{1}$ = 2.18 kΩ and $R_{2}$ = 3.47 kΩ. Each sensor was scanned sequentially, with one complete excitation cycle—comprising a $t^{+}$ and $t^{-}$–phase—allocated per sensor. For each polarity, 100 ADC samples were acquired over a duration of approximately 12.5 ms per phase, resulting in a total cycle time of 25 ms per sensor. To ensure accurate resistance extraction, each plateau was held long enough for the op-amp output to settle. A fixed delay of approximately 1 ms was inserted after each multiplexer switching event before starting ADC sampling, mitigating errors from transient disturbances due to the OP07’s limited slew rate. This ensured that all samples were taken during the stable plateau region. The waveform captures confirm that the output voltages $V_{o}^{+}$ and $V_{o}^{-}$—vary clearly with different $R_{x}$ values and remain well within the OP07’s linear operating range, supporting the system’s ability to perform fast and accurate measurements without analog filtering.

In practice, several hardware design measures were applied to enhance robustness under real-world conditions. First, ADC sampling jitter was minimized by synchronizing data acquisition with the excitation waveform and averaging 100 samples per phase. Second, bias noise was reduced by decoupling the virtual ground ($V_{bias}$) using a local bypass capacitor at the op-amp input. Third, PCB layout was optimized with a solid ground plane and short return paths to minimize ground bounce and common-mode noise. These techniques collectively improved measurement repeatability and noise immunity, particularly in embedded or field-deployed systems. The clearly alternating output plateaus—$V_{o}^{+}$ and $V_{o}^{-}$–corresponded to each excitation phase and displayed distinguishable amplitudes between the two sensor nodes, verifying accurate resistance tracking. The clean waveform transitions confirm correct diode steering, stable excitation, and low crosstalk, supporting the system’s effectiveness for time-resolved multiplexed readout of resistive sensor arrays. The alternating output plateaus $V_{o}^{+}$ and $V_{o}^{-}$ − correspond to the excitation phases $t^{+}$ and $t^{-}$, respectively. To account for the OP07’s limited slew rate and circuit settling behavior, a fixed delay of approximately 1 ms is inserted after each multiplexer switching event before ADC sampling.

In large-scale sensor arrays, practical non-idealities such as increased capacitive loading on long interconnects and leakage currents from multiplexers may affect signal fidelity. These effects can introduce settling delays, slow edge transitions, and minor offsets in the measured plateaus. However, since the proposed method samples only steady-state voltages after switching transients have settled, such errors are inherently reduced. The op-amp’s high input impedance and the use of digital switches with low leakage current (e.g., CD4051B) help minimize static offsets. To further mitigate capacitive artifacts in future high-density implementations, shorter interconnects, shielded traces, and analog switch ICs with lower parasitic capacitance may be employed. Moreover, extending the plateau duration slightly or applying software baseline correction can ensure continued measurement accuracy in dense array configurations.

### 3.2. Uncertain Measurement

**Figure 5:**
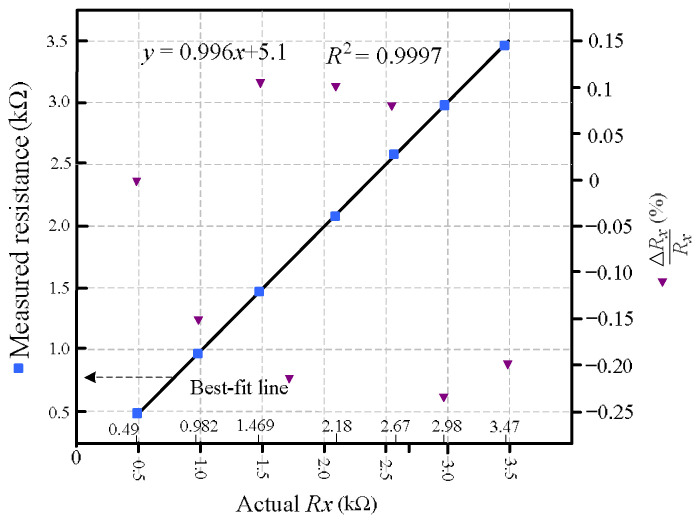
Measured resistance versus actual resistance for various test resistors. Tests were conducted with $R_{WM}$ = $R_{WN}$ = 100 Ω. The blue squares represent measured values with best-fit linearity, while purple triangles indicate relative error $∆R_{x}/R_{x}$. The maximum error was observed to be 0.23%.

In this prototype, the offset compensation pins (VosTrim) of the OP07 operational amplifier were left unconnected. Instead of hardware trimming, a simple one-point software calibration was employed: the system was configured with $R_{x}=0$, and the bias voltage $V_{bias}$ was manually adjusted until the computed resistance output also read zero. This approach does not aim to resolve the op-amp’s input offset voltage directly—typically in the tens of microvolts, which is below the resolution of the 10-bit ADC—but rather to eliminate its systematic effect on the measured differential output quantity $V_{o}^{+}+V_{o}^{-}-2V_{bias}$. Since this differential output is used in the resistance extraction formula, a fixed offset results in a constant baseline shift across all measurements. By aligning the baseline at $R_{x}=0$, the calibration effectively cancels such offsets—including contributions from diode mismatch and op-amp bias asymmetry—without the need for physical trimming circuitry. This method simplifies the system design while maintaining reliable measurement accuracy in low-cost embedded environments. After calibration, a set of known resistors was used to evaluate performance across the target operating range. Eight resistors with nominal values of 0.49 kΩ, 0.982 kΩ, 1.469 kΩ, 2.18 kΩ, 2.67 kΩ, 2.98 kΩ, and 3.47 kΩ were selected, as indicated by the boxed labels along the *x*-axis in Figure 5. These values span typical ranges encountered in practical resistive sensing applications and support evaluation of accuracy at low, mid, and high resistance levels. To emulate remote sensing conditions, the lead-wire resistances were fixed at $R_{WM}$ and $R_{WN}$ = 100 Ω. All resistor values were independently verified using a TECPEL DMM8050 digital multimeter.

The sensor resistance was calculated from the differential output quantity $V_{o}^{+}+V_{o}^{-}-2V_{bias}$ using (3). In Figure 5, the best-fit line represents the linear regression between the measured resistance and the actual resistance values. This line serves two purposes: (i) it confirms the strong linearity achieved by the proposed readout method, and (ii) it demonstrates that parasitic wire and switch resistances introduce no observable gain distortion across the measurement range. The slope of the best-fit line, which remains close to unity, indicates consistent measurement scaling, while the clustering of data points around this line suggests minimal variation across the tested resistance values. The alignment of the slope across different segments—low, medium, and high resistance—reflects uniform behavior of the circuit throughout the range. To quantify this, a statistical analysis was performed, and the coefficient of determination (*R*^2^) between the measured and actual resistance values was computed. The result yielded an *R*^2^ value of 0.9997, indicating an excellent linear fit with negligible deviation. The relative error $∆R_{x}/R_{x}$, plotted on the secondary *y*-axis, remains within ±0.23% for all test points. These results confirm that the proposed circuit enables accurate, wire-resistance-insensitive measurements without requiring complex signal processing or multi-point calibration, even under significant parasitic interconnect conditions.

### 3.3. Robustness to Wire Resistance Variations

**Figure 6:**
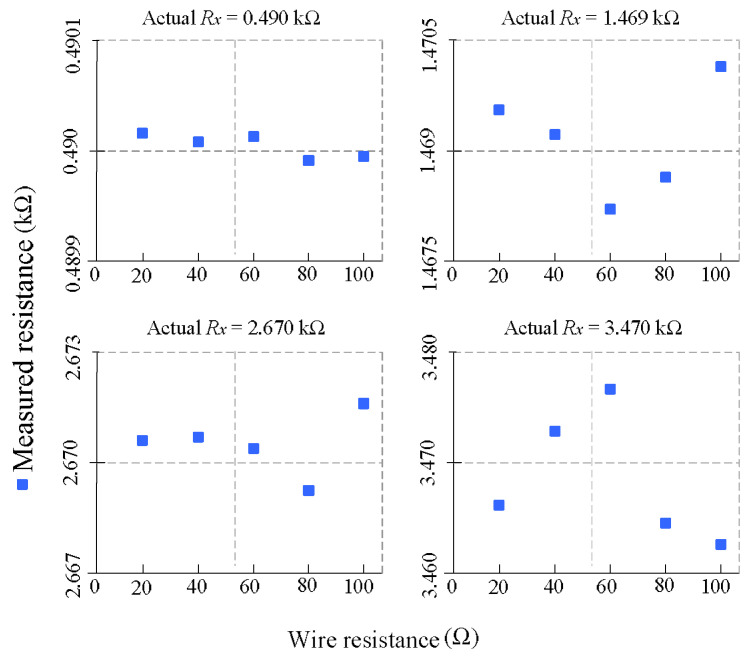
Measured resistance as a function of wire resistance (20–100 Ω) for four different sensor values. All measured values remained within 0.23% of the true resistance across the full range of parasitic wire conditions.

To evaluate the immunity of the proposed method to parasitic lead-wire resistance, additional experiments were conducted by varying the wire resistances $R_{WM}$ and $R_{WN}$ from 20 Ω to 100 Ω in 20 Ω steps. Four different sensor values were tested: 0.49 kΩ, 1.469 kΩ, 2.67 kΩ, and 3.47 kΩ. In each case, the measured resistance was computed from the sampled output voltages using Equation (3) and compared to the actual sensor value. Figure 6 presents the results.

For all tested sensor resistances, the measured values remain remarkably stable across the entire range of wire resistance. No significant deviation or trend is observed, confirming that the differential measurement technique effectively cancels the symmetrical parasitic contributions of wire and switch resistance. The maximum observed deviation across all experiments was less than 0.23%, demonstrating excellent robustness. These results validate the effectiveness of the proposed readout approach in rejecting lead-wire effects without the need for complex compensation or calibration, even in scenarios involving high parasitic resistance such as remote or wearable sensor deployments.

## 4. Discussion

The proposed circuit provides a practical and scalable solution for accurate two-wire resistive sensor readout under long-lead and high-parasitic conditions. Unlike conventional array configurations based on voltage dividers or row–column scanning [16,17,18,19], which often suffer from crosstalk, parasitic paths, and complex analog front ends, this approach uses a single op-amp and diode-steered current paths to isolate each sensor. Only one sensor is activated per cycle, ensuring minimal interference and improved signal integrity without the need for shielding or differential amplifiers. Although the wiring requirement is higher than passive matrix arrays, this trade-off yields a significant benefit in measurement accuracy and robustness. Parasitic effects from long cables and analog switches are inherently canceled through differential sensing, allowing the system to maintain sub-0.3% error across a wide sensor range—even under wire resistance as high as 100 Ω. With minimal analog components and low power needs, the design is suitable for wearable, environmental, or low-cost embedded platforms.

Compared to the method proposed by Reverter [11], which also employs diode-based bidirectional excitation for two-wire resistive sensing, the present system offers superior scalability and dynamic response. Although the approach achieves very high accuracy (approximately 0.005% relative error), it relies on a low-pass filter with a cutoff frequency of 0.16 Hz to extract the averaged op-amp output, which significantly limits the measurement bandwidth and results in a slow response. In contrast, the proposed method directly samples steady-state output voltage plateaus without analog filtering or strict PWM duty-cycle constraints, enabling resistance measurements to be completed within milliseconds and making the system suitable for real-time sensing applications. To further clarify the contributions and practical advantages of the proposed system, Table 2 presents a comparative summary of this method and several representative prior works on resistive sensor array readouts, including [11,16,17,18,19]. The table highlights differences in excitation and readout methods, scalability, crosstalk immunity, hardware complexity, and measurement accuracy. As summarized, the proposed technique strikes a favorable balance between simplicity, accuracy, and scalability—making it well-suited for real-world deployments in compact, multi-sensor, or remote environments. Moreover, because the circuit produces well-defined differential output plateaus centered around a virtual bias point, it is inherently compatible with higher-resolution ADCs (e.g., 12-bit to 16-bit) used in modern embedded platforms. The design’s resilience to noise, combined with digital averaging, makes it highly suitable for integration into low-current, high-precision systems without requiring analog filtering or complex compensation, thereby supporting next-generation sensor technologies in wearable, IoT, or environmental monitoring applications.

Moreover, because the circuit produces well-defined differential output plateaus centered around a virtual bias point, it is inherently compatible with higher-resolution ADCs (e.g., 12-bit to 16-bit) used in modern embedded platforms. The design’s resilience to noise, combined with digital averaging, makes it highly suitable for integration into low-current, high-precision systems without requiring analog filtering or complex compensation, thereby supporting next-generation sensor technologies in wearable, IoT, or environmental monitoring applications. While the system demonstrates strong performance in these areas, one important consideration involves the analog multiplexer (CD4051B). Its on-resistance exhibits a nonlinear dependence on the switched signal level—lowest near mid-supply (≈2.5 V) and increasing toward the supply rails—due to its CMOS pass-transistor structure. This behavior may introduce minor nonlinearities in the resistance extraction process, particularly at extreme $R_{x}$ values where the op-amp output approaches the lower (0 V) or upper (*V*cc) supply limits. Furthermore, since the Arduino’s ADC uses *V*cc as its reference voltage, any variation in the supply can directly impact measurement accuracy. These issues were mitigated in this prototype through careful design, stable power supplies, and a software-based calibration procedure. Nevertheless, future implementations may benefit from precision voltage references, lower $R_{on}$ analog switches, or ratiometric signal processing for enhanced accuracy and robustness.

## 5. Conclusions

This paper presents a scalable op-amp-based readout interface for accurate measurement of remote resistive sensors arranged in a multiplexed matrix. By employing diode-guided bidirectional excitation and analyzing differential op-amp output plateaus, the proposed method effectively eliminates the influence of parasitic resistances—including analog switch on-resistance and long wire leads—without requiring analog low-pass filtering, strict PWM duty cycle, or additional compensation circuitry.

Experimental results confirm that the circuit maintains high measurement accuracy across a wide range of sensor values and parasitic conditions, with a maximum observed error below 0.23% even when the lead-wire resistance is varied up to 100 Ω. Compared to previous works, the method provides a superior balance between accuracy, responsiveness, and implementation simplicity, making it well suited for remote, wearable, or distributed sensor platforms. Future work may explore full matrix implementations with multiple column returns, automatic sensor calibration, and integration with higher-resolution ADCs to further improve performance in ultra-low current applications.

## Figures and Tables

**Table 1:** Experimental Setup and Component Specifications.

Component	Specification/Value
Microcontroller	Arduino Mega2560 (10-bit ADC, 5.01 V measured *V*cc)
Readout amplifier	OP07 precision op-amp
Analog multiplexer	CD4051B (for row selection)
Reference resistor ($R_{ref}$)	8.089 kΩ (10% tolerance in prototype; 0.1% recommended for long-term accuracy)
Op-amp bias voltage ($V_{bias}$)	2.5 V (set using precision resistor divider)
Power supply	Dual ±5 V (for analog front-end)
Sensor emulation resistors	0.49–3.5 kΩ (verified using TECPEL DMM8050 multimeter)

**Table 2:** Comparison of Resistive Sensor Readout Techniques.

Feature/ Reference	Readout Method	Accuracy/ Max Error	Accuracy/ Max Error	Filtering/ Calibration	Crosstalk Handling	Scalability	Comments
Thiswork	Op-amp + diode steering + differential output	0.23%	0.5–3.5 kΩ	No LPF, one-point calibration	Inherently suppressed via symmetric paths	Inherently suppressed via symmetric paths	Compact, parasitic-tolerant, low-power; ideal for embedded sensor arrays
[11], 2023	Op-amp + diode steering + differential output	0.005%	0.06–0.27 kΩ	Requires LPF and precise 50% PWM	N/A (single sensor only)	Low	Ultra-high accuracy; limited to single sensor; response time ~6.3 s (cutoff 0.16 Hz)
[16], 2024	Op-amp + diode + bipolar PWM averaging	~1%	0.5–5 kΩ	Calibration needed	Poor Calibration needed	Moderate	Low hardware cost, but vulnerable to offset and crosstalk
[17], 2023	Passive matrix scan + voltage dividers	0.5–1%	1–10 kΩ	Digital gain adjustment + filtering	Moderate	Moderate	Better linearity than [16]; medium hardware complexity
[18], 2024	Active compensation with DSP	0.06%	0.2–10 kΩ	Multi-point calibration + DSP	Excellent	Moderate	High-end FPGA-based solution; accurate but complex
[19], 2021	Pixel-integrated current source	~<1%	N/A (custom chip)	Multi-point calibration + DSP	Excellent On-chip trimming	Low (per-pixel amp)	High-end FPGA-based solution; accurate but complex

## Data Availability

The data presented in this study are available on request from the Corresponding author.
